# Programming nonreciprocity and harmonic beam steering via a digitally space-time-coded metamaterial antenna

**DOI:** 10.1038/s41598-023-34195-8

**Published:** 2023-05-05

**Authors:** Shaghayegh Vosoughitabar, Chung-Tse Michael Wu

**Affiliations:** Department of Electrical and Computer Engineering, Rutgers, the State University of New Jersey, Piscataway, USA

**Keywords:** Electrical and electronic engineering, Metamaterials

## Abstract

Recent advancement in digital coding metasurfaces incorporating spatial and temporal modulation has enabled simultaneous control of electromagnetic (EM) waves in both space and frequency domains by manipulating incident EM waves in a transmissive or reflective fashion, resulting in time-reversal asymmetry. Here we show in theory and experiment that a digitally space-time-coded metamaterial (MTM) antenna with spatiotemporal modulation at its unit cell level can be regarded as a radiating counterpart of such digital metasurface, which will enable nonreciprocal EM wave transmission and reception via surface-to-leaky-wave transformation and harmonic frequency generation. Operating in the fast wave (radiation) region, the space-time-coded MTM antenna is tailored in a way such that the propagation constant of each programmable unit cell embedded with varactor diodes can toggle between positive and negative phases, which is done through providing digital sequences by using a field-programmable gate array (FPGA). Owing to the time-varying coding sequence, harmonic frequencies are generated with different main beam directions. Furthermore, the space time modulation of the digitally coded MTM antenna allows for nonreciprocal transmission and reception of EM waves by breaking the time-reversal symmetry, which may enable many applications, such as simultaneous transmitting and receiving, unidirectional transmission, radar sensing, and multiple-input and multiple-output (MIMO) beamformer.

## Introduction

Owing to the fact that the magnetic or electric properties can be modulated periodically in both spatial and temporal domain^1^, several space-time modulated architectures have been proposed that can work as a mixer, circulator, transceiver, or nonreciprocal components^1–11^. Very recently, digitally-coded metasurfaces have appeared to be an effective device to control electromagnetic (EM) waves through manipulating the reflection or transmission phase of each programmable unit cell by switching the integrated PIN diode between on “1” and off “0” states^12–14^. By changing coding sequences periodically in the time domain, a space-time coding digital metasurface can be realized to provide interesting applications^15–27^. For example, digitally coded space-time modulation is leveraged to control the pattern of the reflected harmonic waves from the metasurface^15^, break the time reversal symmetry by properly designing the digital coding sequences, thereby enabling nonreciprocal wave reflection and frequency conversion^16^. In a separate study, a dual-channel wireless communication system utilizing a space-time coding digital metasurface is proposed^28^. Furthermore, in a recently published research^29^, a space-time-coding waveguide-integrated metasurface antenna that can control the harmonic patterns independently is realized.

On the other hand, several reconfigurable antennas employing digitlly-coded radiating elements have been reported to enable various functionalities. For example, two planar antenna arrays employing digitally coded radiating elements are proposed to control the radiation beams of the antenna in two dimentional space by designing different coding patterns^30,31^. Furthermore, time modulation has also been applied to the antenna arrays^32^, in which a predetermined periodic sequence can achieve side lobe reduction for the fundamental frequency pattern^33^. Moreover, generated harmonic components can be leveraged to provide physical layer security in wireless communication as well as enable radar detection.^34–45^. Nevertheless, for conventional time modulated antenna arrays, PIN diodes are usually employed as RF switches that decrease the power efficiency due to the power dissipation in their off state^46^.

**Figure 1:**
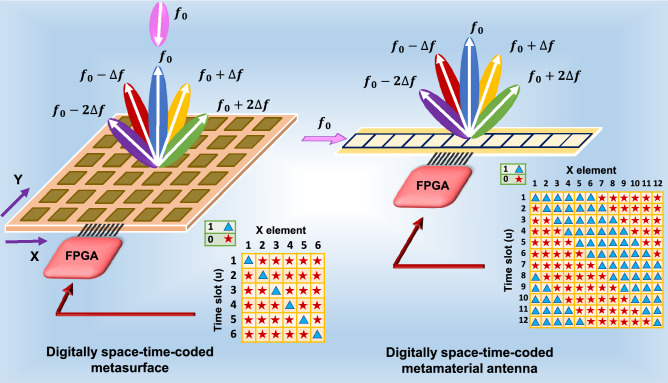
Space-time-modulated metamaterial (ST-MTM) leaky wave antenna as a radiating counterpart of the space-time-modulated metasurface. The space time coding matrices are generated by an FPGA. For the metasurface, the coding sequence is uniform along the Y axis, where “0” and “1” refer to the reflection phase of $0^{\circ }$ and $180^{\circ }$, respectively. For the proposed ST-MTM leaky wave antenna, “0” and “1” refer to a negative and positive phase constant ($\beta$), respectively.

Recently, not only metasurfaces^47^ but also metamaterial antennas have demonstrated their potential for utilization in wireless communication applications. These include directional modulation transmitters^48^, multiple-input multiple-output (MIMO) systems^49^, and wireless body area networks^50^, etc. In this work, we propose a programmable space-time digitally modulated composite right/left-handed (CRLH) metamaterial (MTM) leaky wave antenna^51^ that can be viewed as a radiating counterpart of the space-time-modulated digital metasurface as shown in Fig. 1. To illustrate, MTMs are artificial EM materials with novel effective medium properties that may not be available in nature. One type of MTM antenna is so-called CRLH transmission line leaky-wave antennas exhibiting continuous backfire-to-endfire frequency-dependent beam scanning with a true broadside main beam. Due to the passive nature, conventional CRLH MTM antennas are reciprocal structures operating at the fundamental dominant mode, which cannot separate transmit and receive signals without an external duplexer. To this end, we show a space-time modulated CRLH MTM antenna with digitally programmable unit cells can enable nonreciprocal EM wave transmission and reception. Moreover, harmonic beam controlling can also be achieved through feeding appropriate coding sequences.

In the case of a space-time-modulated metasurface, an incoming signal at $f_0$ frequency is illuminated to the surface, whereas the generated harmonics are reflected to the free space. By manipulating the phase and magnitude distribution of the surface through feeding appropriate periodic sequences to each unit cell, harmonic beamforming capability can be achieved. On the other hand, for our proposed space-time metamaterial (ST-MTM) antenna, a signal at $f_0$ is injected to the antenna input port and the generated harmonics will radiate to the free space. We show theoretically and experimentally that our proposed architecture enables harmonic scanning, simultaneous transmitting and receiving, and nonreciprocal behavior by providing proper spatiotemporal coding sequences for modulating the phase constant of each programmable CRLH MTM unit cell in a periodic fashion. For proof-of-concept realization, varactors are incorporated into each CRLH unit cell to change its phase constant between the positive “state 1” and negative “state 0” values by feeding two specific bias voltages^52^, in which the prespecified periodic sequences are generated by an FPGA and employed as varactors’ control voltages. To the best of the authors’ knowledge, this is the first digitally space-time-coded programmable CRLH MTM antenna exhibiting nonreciprocal and harmonic beamsteering capabilities, which can open up a new paradigm of next-generation intelligent antennas.

## Results

### Formulation of the radiation pattern of a digitally coded space-time modulated metamaterial (ST-MTM) leaky wave antenna

We first consider a programmable CRLH MTM unit cell consisting of interdigital capacitance and shunt-stub inductance with embedded varactor diodes shown in Supplementary Fig. S1A. The absolute value of phase constant ($\beta$) of the designed unit cell multiplied by the unit cell length *p* versus frequency, known as the dispersion curve, for two different bias voltages of the integrated varactors is illustrated in Supplementary Fig. S1B. The frequency region under the air line is regarded as the fast wave region in which the CRLH unit cells can radiate. By changing the varactor bias voltage, one can manipulate the dispersion curve of the MTM unit cell, where the phase constant can alternate its polarity under two bias voltages (Bias 1 and Bias 0) at a given frequency located in the fast wave region.

As such, both positive and negative phase constants can be achieved by simply changing the control voltage from Bias 1 to Bias 0, thereby realizing a binary digital MTM radiating unit cell. We denote that state “1” provides positive $\beta$ and state “0” provides negative $\beta$. A leaky wave antenna can thus be achieved by cascading such digital binary CRLH MTM cells, where the input signal operating in fast wave region radiates out as it propagates along the MTM structure. Moreover, by imposing the time modulation, the phase constant of each unit cell, i.e., $\beta$, changes with time in a periodic manner. By incorporating the array factor approach^53^, in the transmit mode, the radiation pattern of a ST-MTM leaky wave antenna with digital coding can be expressed as:

1 $$\begin{aligned} R(\theta ,t)&= \ S(t)\sum _{n = 1}^{N}{{e^{- \alpha (n - 1)p}e}^{jk_{q}(n - 1)pcos\theta }U_{n}(t)}, \end{aligned}$$

2 $$\begin{aligned} U_{n}(t)&= e^{- j\sum _{m = 1}^{n}\varphi _{m(t)}} = \sum _{u = 1}^{L}{\gamma _{n}^{u}H_{u}(t)}, \end{aligned}$$

3 $$\begin{aligned} H_{u}(t)&= {\left\{ \begin{array}{ll} 1 &{} \frac{(u - 1)T}{L} \le t \le \frac{uT}{L}\\ 0 &{} \text { Otherwise}, \end{array}\right. } \end{aligned}$$

4 $$\begin{aligned} \gamma _{n}^{u}&= \prod _{m = 1}^{n}e^{- j\varphi _{m}^{u}}, \ \varphi _{m}^{u} = \left\{ \begin{matrix} \beta _{0}p,\ \ \text {State}\ 0, \\ \ \ \beta _{1}p,\ \ \text {State}\ 1,\ \\ \end{matrix} \right. \ \text {in}\ \frac{(u - 1)T}{L} \le t \le \frac{T}{L}, \end{aligned}$$

5 $$\begin{aligned} H_{u}(t)&= \sum _{q = - \infty }^{\infty }{a_{u}^{q}e^{\frac{j2\pi qt}{T}}}, \end{aligned}$$

where *S*(*t*) is the input signal with the frequency $f_0$ and the magnitude $S_0$, which is a sinusoidal wave with frequency $f_0$. *N* is the number of CRLH cells of the leaky wave antenna. *p* is length of the unit cell, $\alpha$ is leakage factor, and *L* is length of the sequence in one period ($T=1/\Delta f$). Moreover, $k_q=2\pi (f_0+q\Delta f)/ c$ is the propagation constant for the *q*th radiated harmonic frequency, where *c* is the light speed. Generated harmonics will radiate into the free space with different propagation constants when modulation frequency ($\Delta f$) is not much smaller than the input RF signal frequency ($f_0$). Each period is divided to *L* time slots and the phase shift of the signal reaches to the *n*th unit cell, is a periodic function of time and over the *u*th time slot is defined as (4). After expansion of $H_u(t)$ in the form of Fourier series shown in (5) and substituting it in (1) and doing some manipulations, finally we obtain (see Supplementary for detailed derivation):

6 $$\begin{aligned} R(\theta ,t)&=\sum _{q = - \infty }^{\infty }e^{- j2\pi t(f_{0} - q\mathrm {\Delta }f)}sinc\left( \frac{\pi q}{L}\right) e^{\frac{j\pi q}{L}}\sum _{u = 1}^{L}e^{\frac{- j2\pi qu}{L}}\sum _{n = 1}^{N}\frac{\gamma _{n}^{u}}{L}{S_{0}e^{- \alpha (n - 1)p}e}^{jk_{q}(n - 1)pcos\theta }. \end{aligned}$$

From (6), it can be seen that the radiated patterns entail both radiation at the fundamental frequency ($f_0$) and generated harmonic frequencies ($f_0-q\Delta f$). Therefore, the far-field radiation pattern of the antenna at the *q*th generated harmonic frequency can be expressed as:

7 $$\begin{aligned} R^{TX}_{q}(\theta ) =sinc\left( \frac{\pi q}{L}\right) e^{\frac{j\pi q}{L}}\sum _{u = 1}^{L}{e^{\frac{- j2\pi qu}{L}}\sum _{n = 1}^{N}\frac{\gamma _{n}^{u}}{L}}{S_{0}e^{- \alpha (n - 1)p}e}^{jk_{q}(n - 1)pcos\theta }. \end{aligned}$$

On the other hand, in the receive mode, assuming that the signal at $f_0$ is illuminated to the digital ST-MTM antenna from the $\theta$ angle, and generated harmonics are received from the same port of signal injection in the transmit mode, these patterns can be written as: (see Supplementary for detailed derivation):

8 $$\begin{aligned} R^{RX}_{q}(\theta ) =sinc\left( \frac{\pi q}{L}\right) e^{\frac{j\pi q}{L}}\sum _{u = 1}^{L}{e^{\frac{- j2\pi qu}{L}}\sum _{n = 1}^{N}\frac{\gamma _{n}^{u}}{L}}{S_{0}e^{- \alpha (n - 1)p}e}^{jk_{0}(n - 1)pcos\theta }. \end{aligned}$$

**Figure 2:**
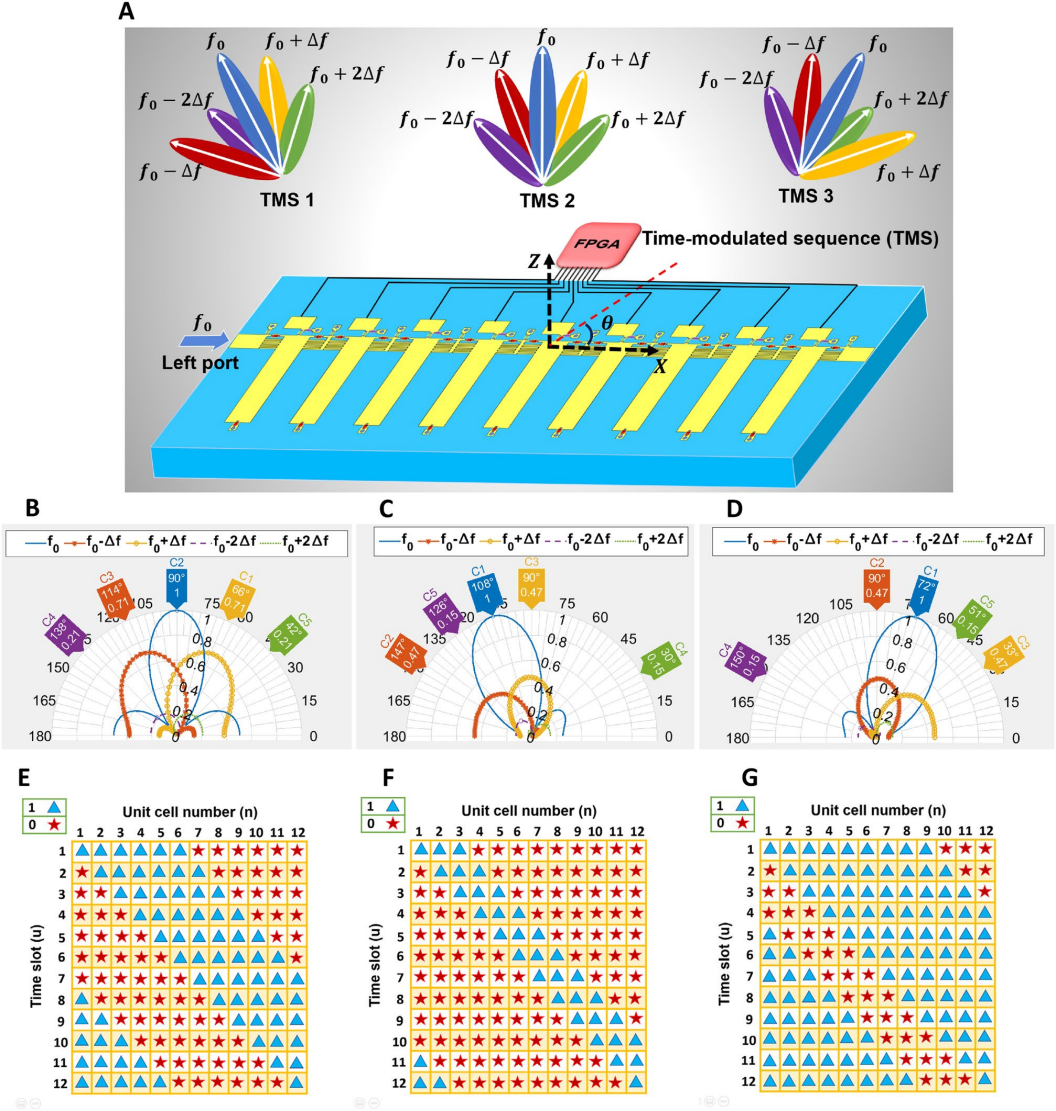
(**A**) Programmable harmonic beam scanning of the proposed ST-MTM antenna with different space-time coding sequences. Simulated normalized harmonic patterns (dB) when the signal is injected from the left port. (**B**) for sequence 111111000000. (**C**) for sequence 111000000000. (**D**) for sequence 111111111000. State of each unit cell in different time slots in one period for (**E**) figure (**B**). (**F**) figure (**C**). (**G**) figure (**D**).

The only difference between the Eqs. (8) and (7) is replacing $k_q$ with $k_0=2\pi f_0/c$, since the incident signal operates at $f_0$ in the free space and harmonics are generated when the signal is traveling inside the ST-MTM antenna. The pattern of the harmonic frequencies can be controlled by properly feeding the periodic sequence to each unit cell, thereby enabling several functionalities such as harmonic beam scanning, simultaneous transmitting and receiving, and nonreciprocity. The schematic of the proposed ST-MTM antenna consisting of programmable CRLH unit cells is illustrated in Fig. 2A.

### Programmable harmonic beam scanning

As also shown in Fig. 2A, the proposed ST-MTM antenna can exhibit dynamic harmonic beam steering, in which the fundamental and harmonic radiation patterns are plotted in Fig. 2B–D when the signal at the fundamental tone is injected from the left port with $N =L =12$. According to the simulated dispersion diagram of the designed programmable CRLH unit cell in Bias 0 and Bias1 states shown in Supplementary Fig. S1B, the negative and positive phase shift values ($\beta _{0}p\ ,\beta _{1}p$) around 2.05 GHz located in the fast wave are 24 and − 24 degrees, respectively. In Fig. 2B the sequence is 111111000000 for the 12 cells in the first time slot and then it is circulated one bit backward for the next time slots as illustrated in Fig. 2E to set the main beam direction of fundamental frequency in broadside. As observed, harmonic beam scanning is achieved by using this sequence, i.e. each harmonic frequency component has its own main beam direction. By changing the sequence to 111000000000 and 111111111000, the main beam of the fundamental frequency and harmonic components can be shifted to the left and right side, respectively, as shown in Fig. 2C,D. Figure 2F,G depict the space time coding matrix corresponding to the patterns shown in Fig. 2C,D (see Supplementary Fig. S4 for more results showing harmonic beam scanning). The resulting harmonic beam scanning can be utilized in various applications, including multipoint communication purposes or radar sensing systems for multi target detection.

### Simultaneous transmitting and receiving

**Figure 3:**
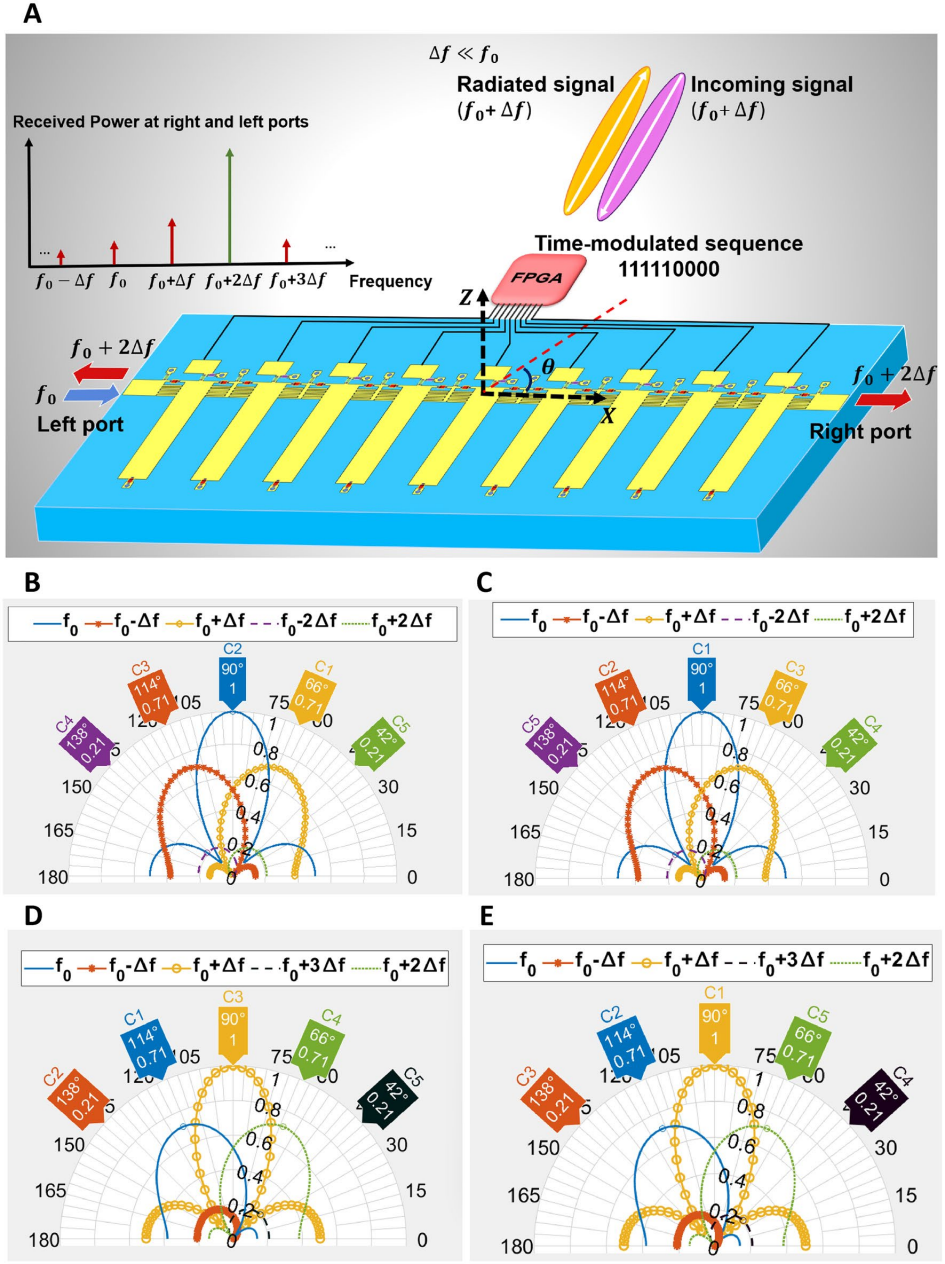
(**A**) Simultaneous transmitting and receiving by ST-MTM antenna when ($\mathrm {\Delta }f \ll f_{0}$). Simulated normalized harmonic patterns (dB) for sequence 111111000000. (**B**) when the signal is injected from the left port at $f_0$. (**C**) when the signal is injected from the right port at $f_0$. (**D**) when the signal is illuminated at $f_0+\Delta f$ and harmonics are received from the left port. (**E**) when the signal is illuminated at $f_0+\Delta f$ and harmonics are received from the right port.

The proposed digitally coded ST-MTM antenna is essentially a 2-port structure and can transmit and receive the information simultaneously as depicted in Fig. 3A. Figure 3B illustrates the case where the fundamental tone signal is injected from the left port, which has the same patterns as Fig. 2B.

Figure 3C illustrates the patterns for injecting the signal from the right port with the same space-time sequence used in Fig. 3B. According to (7) and (8), it can be observed that if $\mathrm {\Delta }f \ll f_{0}$ for each harmonic frequency, the far-field pattern when the signal with $f_{0}$ frequency is injected from the left/right port is the same as the received power of that harmonic in different angles from the same port when the signal is illuminated at $f_{0}$ frequency to the ST-MTM antenna. Therefore, we can consider Fig. 3C patterns for illuminating the signal at $f_{0}$ frequency from different angles and receiving the fundamental signal and harmonic components from the right port. As clearly observed in Fig. 3B,C the patterns are the same for each harmonic frequency when the signal with frequency $f_{0}$ is injected from the left and when the signal is illuminated to the ST-MTM antenna, and power levels are recorded from the right port.

Considering this interesting property, the idea of simultaneous transmitting and receiving can be made feasible as illustrated in Fig. 3A. Assuming in the transmit mode, the signal is injected at $f_{0}$ frequency from the left port, a transceiver located in the main beam direction of $f_{0} + \mathrm {\Delta }f$ pattern can receive the signal at $f_{0} + \mathrm {\Delta }f$ and transmit another signal with this frequency at the same time. In this case under the assumption $\mathrm {\Delta }f \ll f_{0}$ , the generated normalized harmonic patterns extracted from the left and right ports are shown in Fig. 3D,E, respectively. According to these figures, $f_{0} + \mathrm {\Delta }f + \mathrm {\Delta }f$, which is the first harmonic in the receive mode, will be the dominant harmonic frequency in this direction and propagates to both right and left ports. As such, the received information can be extracted from the left or right port at $f_{0} + 2\mathrm {\Delta }f$.

### Nonreciprocal behavior of digitally coded ST-MTM antenna

**Figure 4:**
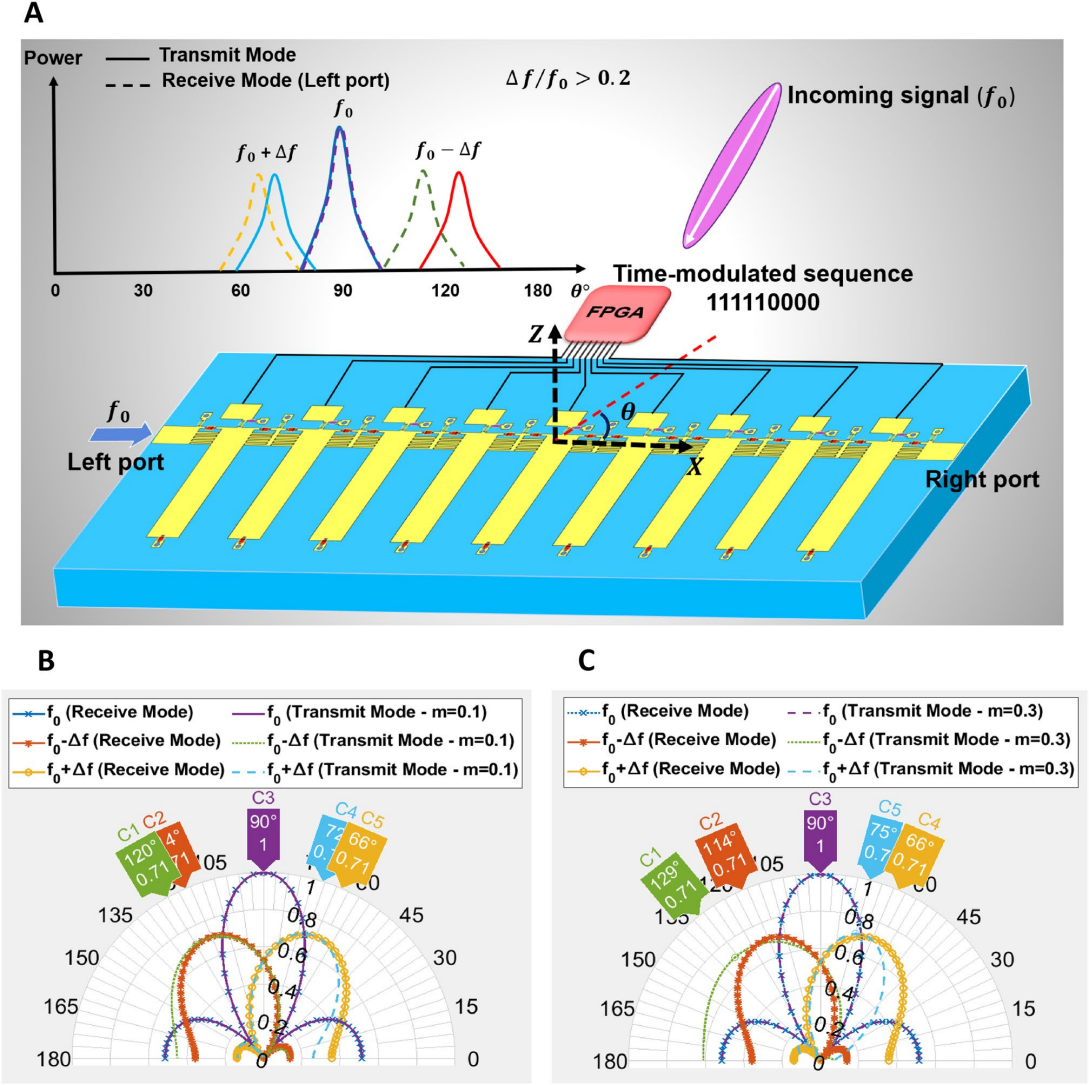
(**A**) Nonreciprocal behavior for transmission and reception of the signal at harmonic frequencies when $\mathrm {\Delta }f/f_{0} > 0.2$. Simulated normalized harmonic patterns in transmit mode and receive mode (dB). (**B**) for $\mathrm {\Delta }f/f_{0} = 0.1$. (**C**) for $\mathrm {\Delta }f/f_{0} = 0.3$.

Based on (7) and (8), when the modulation frequency ($\mathrm {\Delta }f$) is not much smaller than the fundamental frequency ($f_{0}$), the main beam direction of harmonic patterns in the transmit mode will be different than their maximum power direction extracted from the same port in the receive mode shown in Fig. 4A. Figure 4B,C. illustrate the transmission and reception patterns for different modulation frequencies. According to the results, increasing $\mathrm {\Delta }f/f_{0}$ leads to increasing the difference between the main beam direction of transmission and reception patterns of each harmonic frequency. This property depicts the nonreciprocal behavior of the ST-MTM antenna for $m = \mathrm {\Delta }f/f_{0} > 0.2$.

Moreover, when $f_{0}$ is injected from the left port in transmit mode, consider the main beam direction of generated harmonic pattern with $f_{0} + \mathrm {\Delta }f$ frequency in $\theta _{1} = 66{^\circ }$ shown in Fig. 3B. In the receive mode under the assumption $\mathrm {\Delta }f \ll f_{0}$, if a signal with $f_{0} + \mathrm {\Delta }f$ frequency is illuminated to the ST-MTM antenna in different angles, the harmonic patterns extracted from left port are like Fig. 3D by considering $f_{0} + \mathrm {\Delta }f$ as fundamental frequency. As such, illuminating the signal at $f_{0} + \mathrm {\Delta }f$ frequency from $\theta _{1}$ angle to the antenna leads to reception of a very small power at $f_{0}$ frequency from the left port, thereby exhibiting nonreciprocal operation.

### Experimental verification

**Figure 5:**
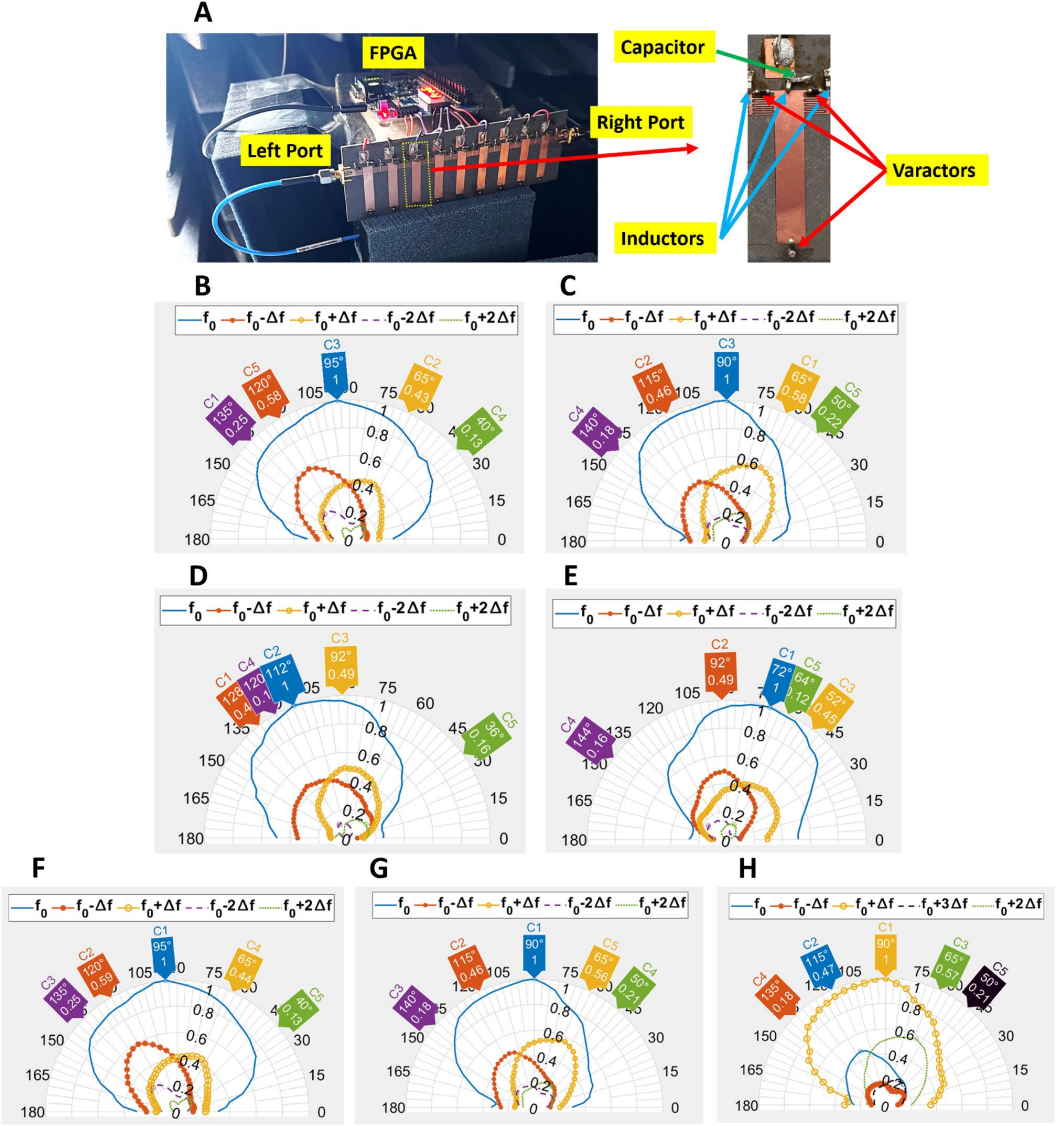
(**A**) Fabricated prototype of the proposed ST-MTM antenna consisting of 9 programmable CRLH unit cells with the close-up view of the MTM unit cell. (**B**) Measured normalized harmonic patterns (dB) in the transmit mode with sequence 111110000 when the signal is injected from the left port. (**C**) The signal is injected from the right port. (**D**) The signal is injected from the left port with sequence 110000000. (**E**) sequence 111111100. (**F**) Receive mode: The ST-MTM antenna is illuminated by a horn antenna at $f_0$ for sequence 111110000, and signals are received from the left port. (**G**) from the right port. (**H**) The ST-MTM antenna is illuminated by a horn antenna at $f_0+\Delta f$, and the signals are received from the right port for sequence 111110000. In all cases, $f_0$ is 2.05 GHz and $\Delta f$ is 1.58 MHz.

As illustrated in Fig. 5A, a prototype of the proposed digitally space-time-coded MTM antenna with 9 unit cells is fabricated to validate the aforementioned concepts. Each unit cell includes three varactors sharing a common bias voltage. According to the dispersion diagram of the unit cell around 2.05 GHz the phase constant can toggle between positive and negative values by changing the bias of varactors from 1 to 0 state. The number of time slots (*L*) are considered 9 (same as the number of unit cells), and the sequence in the first time slot is shifted by one bit for the next time slot by using an FPGA shown in Fig. 5A. The modulation frequency we apply here is 1.58 MHz, which is much smaller than the fundamental frequency ($\mathrm {\Delta }f \ll f_{0}$). It is worth mentioning that when designing a tunable unit cell with components such as varactors, the switching speed of the varactor should be faster than $L\Delta f$ to ensure effective toggling of $\beta$ between “0” and “1” states as per the designed time-modulated sequence. Additionally, the capability of the FPGA to generate a periodic signal for driving the varactors, based on its clock frequency, is another factor that may impose limitations on the achievable modulation frequency.

Measured *S*-parameters of the sample for different time-modulated sequences are plotted in Supplementary Fig. S2, 3, which depict good $S_{11}$ and $S_{22}$ around 2 GHz. In the transmit mode, a signal with a frequency of $f_{0} = 2.05$ GHz is injected from the left port of the ST-MTM antenna, and a reference antenna located in the far-field region of the ST-MTM antenna receives the fundamental and harmonic frequencies in different angles. Fig. 5B,C show the normalized measured radiation patterns for sequence 111110000 when the signal is injected from the left and right port, respectively. Based on the aforementioned results shown in Fig. 3, it is expected the main beam directions of fundamental and harmonic frequencies for both left- and right-port excitation should be the same, as can be observed in Fig. 5B,C. To verify the harmonic beam scanning, in Fig. 5D we change the sequence to 110000000, whereas in Fig. 5E the sequence is 111111100, with the signal injected from the left port. As can be seen clearly, with different sequences, the main beam directions of the fundamental and harmonic tones can be steered.

Moreover, Fig. 5F,G depict the measured patterns in the receive mode with the sequence 111110000, when the signal with a frequency of $f_{0}$ is illuminated from a reference horn antenna to the ST-MTM antenna at different angles, in which the received spectral components are collected from the left and right port, respectively. The received signals are then normalized to the maximum of the fundamental frequency. It is also expected that the harmonic patterns for the transmit mode and receive mode should be the same, when excited at $f_{0}$, under the condition $\mathrm {\Delta }f \ll f_{0}$, which can be clearly verified from the measured patterns. Furthermore, we then change the illuminated signal frequency to 2.05 GHz$+ \Delta f$, where $\mathrm {\Delta }f$ is 1.58 MHz. The received patterns from the right port are shown in Fig. 5H, where it can be seen that the main beam of 2.05 GHz $+ \Delta f$ is now at the broadside.

It is noticed that the simulated results are based on the antenna with 12 unit cells. Nevertheless, due to the fabrication limitations, we demonstrate a ST-MTM antenna with 9 cells instead. A larger number of MTM unit cells will result in increasing the directivity of the antenna with narrower main beams as can be observed in Fig. 2. While the simulation and measurement results agree with each other, there are some discrepancies in the main beam direction of the fundamental and harmonic frequency patterns, mainly resulting from the difference in unit cell numbers as well as the propagation constant values of the two bias states between the measurement and the full-wave EM simulation. It is noted a small change in the bias voltages come from the FPGA may lead to some deviation of the propagation constant in the two states (0 and 1).

According to the measured results shown in Fig. 5, simultaneous transmitting and receiving is possible based on the proposed approach illustrated in Fig. 3A, where $\mathrm {\Delta }f \ll f_{0}$ for our proposed ST-MTM antenna. On the other hand, the modulation frequency cannot be more than 5.5 MHz due to the hardware limitation of FPGA in the measurement, i.e. the cases when $\mathrm {\Delta }f/f_{0} > 0.1.$

## Methods

For the theoretical results, MATLAB is used to plot the far field pattern of the fundamental and harmonic frequencies based on the derived equations. For the programmable unit cell design, HFSS as a full wave simulator, is utilized to design the MTM unit cell with dispersion diagrams shown in Supplementary Fig. S1B. The length of the unit cell is around 1.5 cm. In the simulation, the varactors are modeled using a series RLC circuit, where R$= 4.8$ $\ \Omega$, L= 0.7 nH, and C varies with respect to the bias voltages. For the Bias “0” state the capacitor value is 1.05 pF and for Bias “1” state it is 2.1 pF. The prototype is simulated and fabricated on a RO5870 substrate with a dielectric constant of 2.33 and thickness of 1.57 mm. The varactors are SMV2019 from Skyworks, where the capacitance range varies from 2.2 pF at a bias voltage of 0 V to 0.3 pF at 20 V. It is noted 0.1 V and 3 V can provide our design with the required positive and negative phase constants, respectively.

Basys 3 FPGA board is used to provide 9 digital outputs for the control bias voltages of each unit cell. The low-level output voltage is around 50 mV, whereas the high-level one is around 3.2 V, which can provide us the 0 and 1 states for the predetermined sequences. The period of the time coding sequence is selected to be 0.63 µs, resulting in a modulation frequency of 1.58 MHz. The radiation pattern measurements are conducted inside a microwave anechoic chamber. A reference horn antenna is used as a transmitter when the prototype operates in the receive mode, and as a receiver when the prototype operates as a transmitter. A signal generator is utilized to provide a signal in the frequency of around 2 GHz. By using a spectrum analyzer, the received signals at the fundamental and harmonic frequencies are collected from the reference horn antenna in the transmit mode, as well as from the left and right port of the prototype in the receive mode when illuminated from different angles.

## Discussion

In this paper, we propose a digitally space-time-coded MTM antenna exhibiting harmonic beam scanning and nonreciprocity behavior, which can be used for simultaneous transmitting and receiving. The phase constant of each varactor-embedded programmable CRLH unit cell changes between positive (state 1), and negative (state 0) values based on a predetermined sequence in a periodic fashion in both space and time domain. In so doing, harmonic beam scanning can be realized by simply changing the programming coding sequences, which may lead to various applications including MIMO communication and radar detection.

Moreover, nonreciprocal transmission and reception of harmonic waves can be achieved by the proposed ST-MTM antenna. When $\mathrm {\Delta }f \ll f_{0}$, the generated harmonic signal at $f_{0} + \mathrm {\Delta }f$ will radiate towards a specific direction in the transmit mode, while in the receive mode, by illuminating a signal at $f_{0} + \mathrm {\Delta }f$ to the ST-MTM antenna at the same direction, the dominant generated harmonic signal becomes $f_{0} + 2\mathrm {\Delta }f$. Leveraging such nonreciprocal property, simultaneous transmitting and receiving can therefore be realized by transmitting the signal at $f_{0}$, whereas the information of the receiving signal can be extracted at the frequency of $f_{0} + 2\mathrm {\Delta }f$.

On the other hand, when the modulation frequency is not much smaller than the fundamental frequency, different main beam directions of harmonic waves for transmit and receive patterns can be observed when injecting or illuminating the antenna at the same frequency. A prototype with 9 programmable MTM unit cells is fabricated, in which the experimental results validate the proposed concept of the ST-MTM antenna.

## Supplementary Information

Supplementary Information.

## Data Availability

The data that support the findings of this study are available from the corresponding author upon reasonable request.
