# Supplementary material for: Programming nonreciprocity and harmonic beam steering via a digitally space-time-coded metamaterial antenna
Source: Sci Rep. 2023 May 5;13:7338. doi: 10.1038/s41598-023-34195-8 (PMC10163271; doi:10.1038/s41598-023-34195-8)
Supplement: Supplementary file 1 — Supplementary Information. [file 41598_2023_34195_MOESM1_ESM.pdf]

Supplementary Information for

Programming nonreciprocity and harmonic beam steering via a digitally space-time-coded metamaterial antenna

Shaghayegh Vosoughitabar and Chung-Tse Michael Wu
Corresponding author: Chung-Tse Michael Wu (Email: ctm.wu@rutgers.edu)

Far-field radiation pattern derivation in the transmit mode. For expansion of $U_n(t)$ in the form of Fourier series, we start from $H_u(t)$:

$$H_u(t) = \sum_{q=-\infty}^{\infty} a_u^q e^{j2\pi q\Delta f t} \tag{1}$$

$$U_n(t) = \sum_{u=1}^{L} \gamma_n^u H_u(t) = \sum_{u=1}^{L} \gamma_n^u \sum_{q=-\infty}^{\infty} a_u^q e^{j2\pi q\Delta f t} = \sum_{q=-\infty}^{\infty}\sum_{u=1}^{L} \gamma_n^u a_u^q e^{j2\pi q\Delta f t} = \sum_{q=-\infty}^{\infty} b_u^q e^{j2\pi q\Delta f t} \tag{2}$$

So, b_u^q is the Fourier series coefficient of the periodic function $U_n(t)$ and is obtained as follows:

$$b_u^q = \sum_{u=1}^{L} \gamma_n^u a_u^q = \sum_{u=1}^{L} \frac{\gamma_n^u}{T_0} \int_{\frac{(u-1)T}{L}}^{\frac{uT}{L}} e^{-j2\pi q\Delta f t} dt = \sum_{u=1}^{L} \gamma_n^u f_0 \frac{e^{-j2\pi q\Delta f t}}{-j2\pi q\Delta f}\Bigg|_{\frac{(u-1)T_0}{L}}^{\frac{uT_0}{L}} \tag{3}$$

$$= \sum_{u=1}^{L} \frac{\gamma_n^u}{-2j\pi q}\left[e^{\frac{-j2\pi qu}{L}} - e^{\frac{-j2\pi q(u-1)}{L}}\right] = \sum_{u=1}^{L} \frac{\gamma_n^u}{-2j\pi q} e^{\frac{-j\pi q(2u-1)}{L}} \sin\left(\frac{\pi q}{L}\right) * (-2j) \tag{4}$$

$$= \sum_{u=1}^{L} \frac{\gamma_n^u}{L} e^{\frac{-j\pi q(2u-1)}{L}} sinc\left(\frac{\pi q}{L}\right) = e^{\frac{j\pi q}{L}} sinc\left(\frac{\pi q}{L}\right) \sum_{u=1}^{L} e^{\frac{-j2\pi qu}{L}} \sum_{n=1}^{N} \frac{\gamma_n^u}{L} \tag{5}$$

By substituting this expression in the far field radiation pattern, we have:

$$R(\theta,t) = S(t)\sum_{n=1}^{N} e^{-\alpha(n-1)p} e^{jk_q(n-1)pcos\theta} U_n(t) \tag{6}$$

$$= e^{j2\pi f_0 t} \sum_{q=-\infty}^{\infty} e^{j2\pi q f_0 t} S_0 e^{-\alpha(n-1)p} e^{jk_q(n-1)pcos\theta} sinc\left(\frac{\pi q}{L}\right) e^{\frac{j\pi q}{L}} \sum_{u=1}^{L} e^{\frac{-j2\pi qu}{L}} \sum_{n=1}^{N} \frac{\gamma_n^u}{L} \tag{7}$$

$$= \sum_{q=-\infty}^{\infty} e^{-j2\pi t(f_0 - q\Delta f)} sinc\left(\frac{\pi q}{L}\right) e^{\frac{j\pi q}{L}} \sum_{u=1}^{L} e^{\frac{-j2\pi qu}{L}} \sum_{n=1}^{N} \frac{\gamma_n^u}{L} S_0 e^{-\alpha(n-1)p} e^{jk_q(n-1)pcos\theta} \tag{8}$$

Therefore, equation (4) is obtained which is equation (6) in the paper.

Formulation of patterns in the receive mode. In the receive mode, a signal with frequency f_0 is illuminated to the ST-MTM antenna. The fundamental and harmonic patterns are received from the left port.

To illustrate, the signal radiated from the nth unit cell in the transmit mode can be written as:

$$R_n^{TX}(\theta,t) = S(t) e^{-\alpha(n-1)p} e^{jk_q(n-1)pcos\theta} U_n(t), \;\; U_n(t) = e^{j\sum_{m=1}^{n}\varphi_{m(t)}} = \sum_{u=1}^{L}\prod_{m=1}^{n} e^{-j\varphi_m^u} H_u(t) \tag{9}$$

In this case, the signal is injected from the left port and reaches to the nth unit cell after passing from the previous $(n-1)$ cells which leads to $j\sum_{m=1}^{n}\varphi_{m(t)}$ phase shift. Then some portion of it radiates to the free space.

On the other hand, in the receive mode after capturing the signal through nth unit cell it travels toward left and right ports. The portion of it that travels toward the left port, passes from the same $(n-1)$ cells and reaches to the left port. In this case, the phase shift will be $j\sum_{m=1}^{n}\varphi_{m(t)}$ which is the same as phase shift in transmit mode. As such, the signal received by the nth unit cell which then reaches to the left port can be written as:

$$R_n^{RX}(\theta,t) = S(t) e^{-\alpha(n-1)p} e^{jk_0(n-1)pcos\theta} U_n(t), \;\; U_n(t) = e^{j\sum_{m=1}^{n}\varphi_{m(t)}} = \sum_{u=1}^{L}\prod_{m=1}^{n} e^{-j\varphi_m^u} H_u(t) \tag{10}$$

The only difference of equation (5) with (6) is replacing k_q with $k_0 = 2\pi f_0 / c$.

Radiation efficiency of the ST-MTM antenna. By defining the radiation efficiency as the ratio of the radiated power by the antenna to the input power accepted by the antenna, the radiation efficiency of a leaky wave antenna can be obtained as[1–3]:

$$\eta = \frac{\alpha_r}{\alpha_t}\left(1 - e^{-2\alpha_t l}\right) , \quad \alpha_t = \alpha_r + \alpha_l , \quad \alpha_t = \frac{-ln|S_{21}|}{l}, \tag{11}$$

where α_r is the leakage rate, α_l is the transmission loss rate, and l is the length of the leaky wave antenna. Considering this, the radiation efficiency in our proposed digitally space-time-coded metamaterial antenna depends on the varactor loss (for SMV2019 varactors, $R = 4.8$ Ohm), substrate loss (RO5870 is a low loss substrate with the loss tangent of 0.0012 at 10 GHz), and leakage rate. According to our measurement under the time-modulated case for different sequences, $|S_{21}|$ is almost the same as the static case (i.e., no time modulation, using the same digitally coded sequences as the first time slot in the time-modulated case). The only difference is that in the static case, the radiated power is at f_0 frequency, while in the time-modulated case, radiations occur across the harmonic frequencies $f_0 + q\Delta f$. Considering the similar insertion loss between the static and time modulated case, according to the simulation for the static case we have:

$$|S_{21}|_{antenna} \approx -14\ dB \ \rightarrow \ |S_{21}|_{antenna} = 0.199 \ \rightarrow \ \alpha_t = \frac{-ln(0.199)}{0.14} = 11.5\ (\frac{Np}{m}) \tag{12}$$

$$\eta = \frac{\alpha_r}{\alpha_t}\left(1 - e^{-2\alpha_t l}\right) = \frac{\alpha_r}{\alpha_t}\left(1 - |S_{21}|^2\right) \tag{13}$$

In order to obtain α_r, in the simulation we set the series R for the varactors equal to 0.01 Ohm and then 4.8 Ohm in the unit cell (unit cell under bias 1 or 0). Around 2 GHz we obtain:

$$R = 0.01\ Ohm \rightarrow \ |S_{21}|_{unitcell} \approx -0.2\ dB\ , \ \ R = 4.8\ Ohm \ \rightarrow \ |S_{21}|_{unitcell} \approx -1.4\ dB \tag{14}$$

It can therefore be observed that most of the loss results from the series resistance of the varactors.

$$\alpha_l = \frac{-ln(10^{(-1.2/20)})}{p} = \frac{-ln(0.871)}{0.015} = 9.2 \tag{15}$$

$$\eta = \frac{11.5 - 9.2}{11.5}(1 - (0.199)^2) \approx 19\% \tag{16}$$

As such, the main factor that decreases the radiation efficiency is the varactor's loss. Choosing a varactor with a low series resistance would increase the radiation efficiency drastically. Moreover, increasing the number of the unit cells (length of the antenna) will also increase the radiation efficiency. Our prototype has only 9 unit cells (due to the fabrication limitations).

Although in the simulation $|S_{21}|$ is around -14 dB, in the measurement (in the static or time modulated case), it is around -24 dB. This means there are more losses in the fabricated prototype due to the additional varactors' losses, soldering effects, as well as other ohmic and dielectric losses.

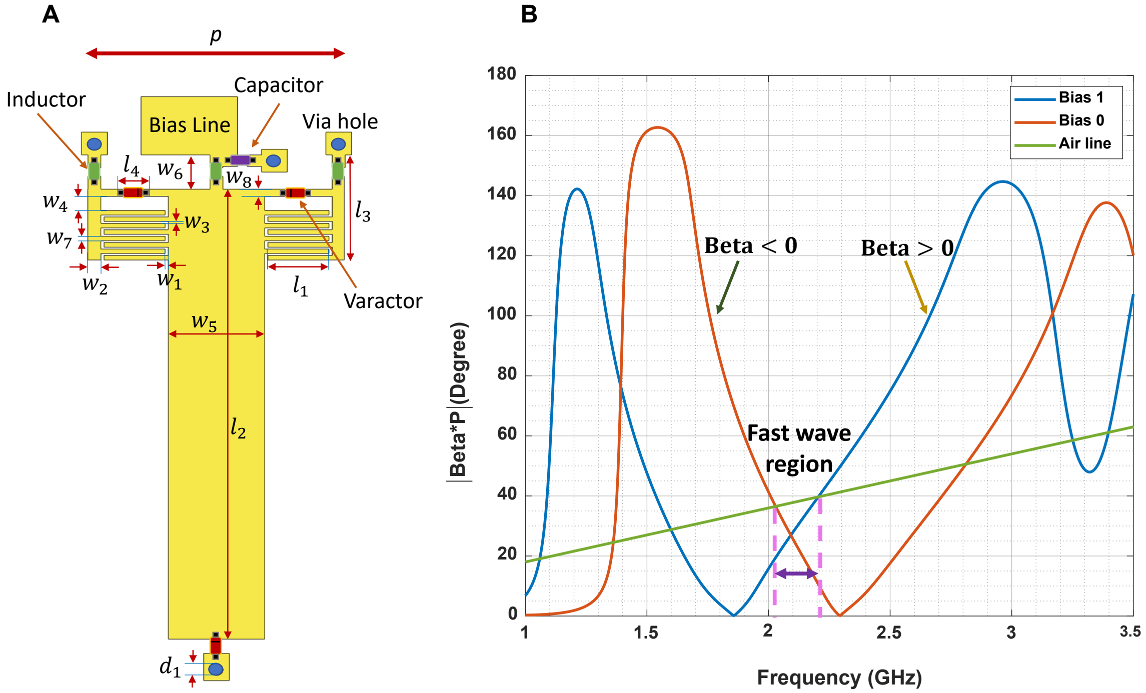

Fig S1. (A)Tunable unit cell schematic. The Dimensions (millimeters) are: $w_1 = 0.2$, $w_2 = 0.8$, $w_3 = 0.15$, $w_4 = 1$, $w_5 = 6$, $w_6 = 2.4$, $w_7 = 0.3$, $w_8 = 0.5$, $l_1 = 3.8$, $l_2 = 31.5$, $l_3 = 7.4$, $l_4 = 1.8$, $d1 = 0.8$. (B) Simulated Dispersion curves for two states of varactor Bias.

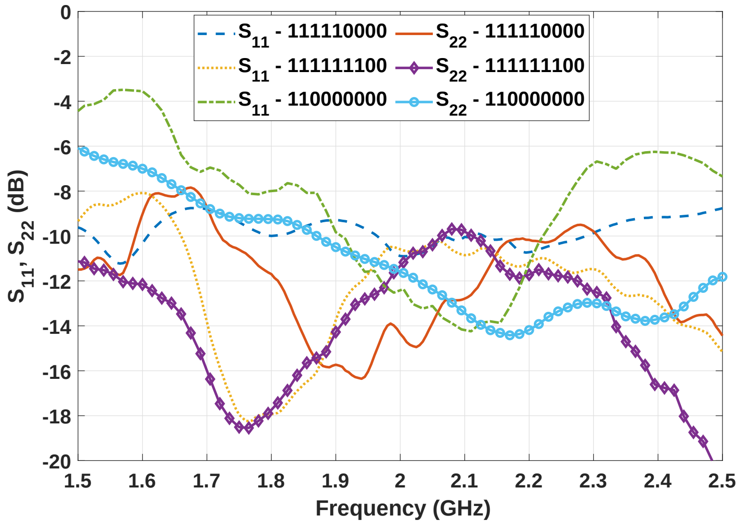

Fig S2. Measured S_{11}, S_{22} of the fabricated ST-MTM antenna in the time-modulated case for the shown sequences in the first time slot.

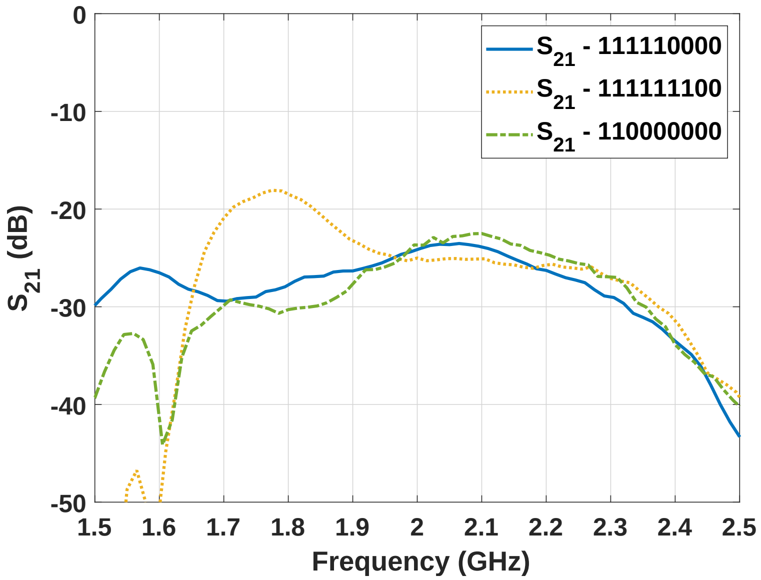

Fig S3. Measured S_{21} of the ST-MTM antenna in the time-modulated case for the shown sequences (in the first time slot).

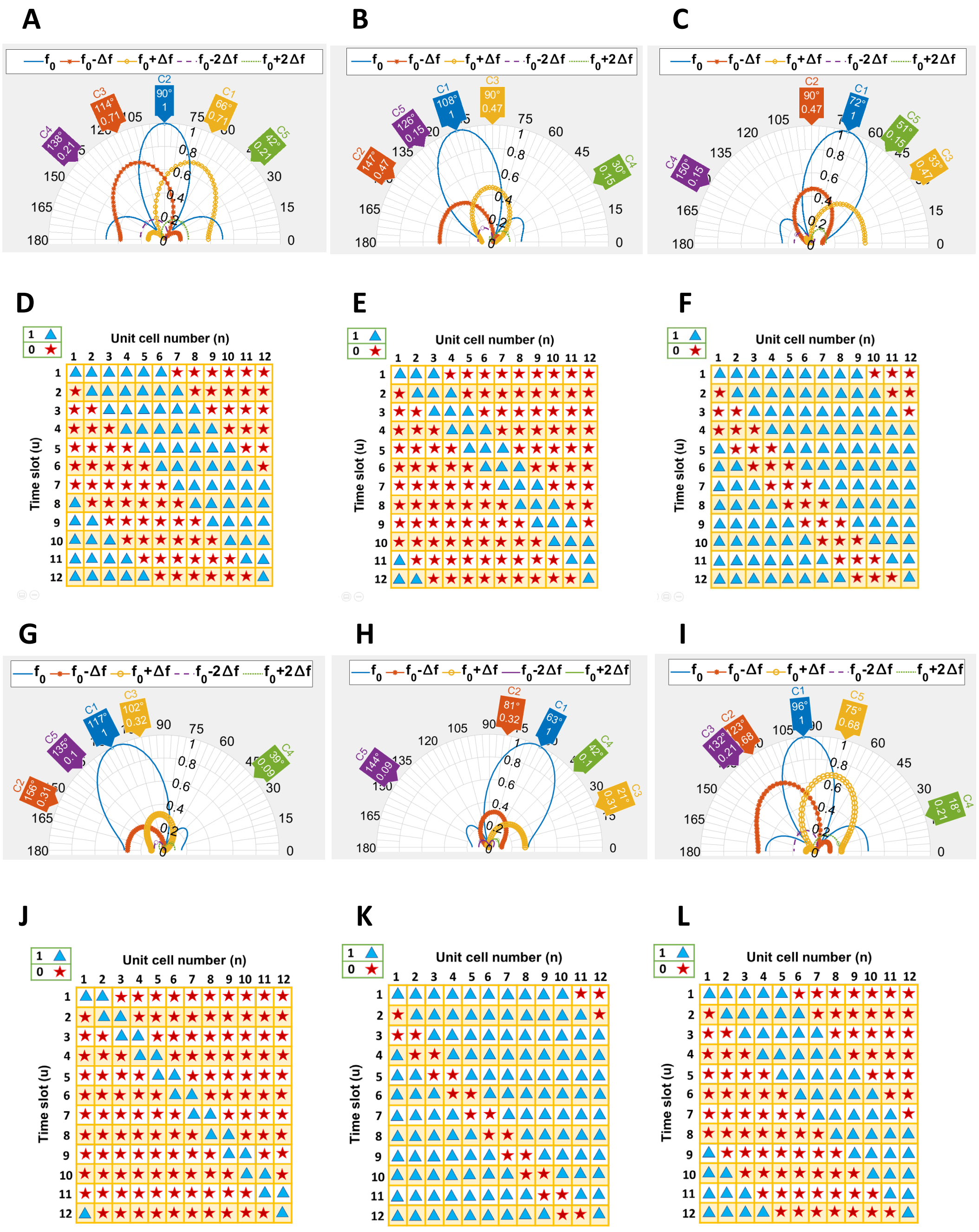

Fig S4. Programmable harmonic beam scanning of the proposed ST-MTM antenna with different space-time coding sequences. Simulated normalized harmonic patterns (dB) when the signal is injected from the left port. (A) for sequence 111111000000. (B) for sequence 111000000000. (C) for sequence 111111111000. State of each unit cell in different time slots in one period for (D) figure A. (E) figure B. (F) figure C. Simulated normalized harmonic patterns (dB) when the signal is injected from the left port. (G) for sequence 110000000000. (H) for sequence 111111111100. (I) for sequence 111110000000. State of each unit cell in different time slots in one period for (J) figure G. (K) figure H. (L) figure I.

References

1. Jackson, D. R., Caloz, C. & Itoh, T. Leaky-wave antennas. *Proc. IEEE* **100**, 2194–2206, DOI: 10.1109/JPROC.2012.2187410 (2012).

2. Gómez-Tornero, J. L., Goussetis, G. & A.Álvarez-Melcón. Correction of dielectric losses in practical leaky-wave antenna designs. *J. Electromagn. Waves Appl.* **21**, 1025–1036, DOI: 10.1163/156939307781749731 (2007). https://www.tandfonline.com/doi/pdf/10.1163/156939307781749731.

3. Itoh, T. & Caloz, C. *Electromagnetic metamaterials: transmission line theory and microwave applications* (John Wiley & Sons, 2005).
